# Tumour-induced apoptosis in human mesothelial cells: a mechanism of peritoneal invasion by Fas Ligand/Fas interaction

**DOI:** 10.1038/sj.bjc.6601635

**Published:** 2004-03-30

**Authors:** R M Heath, D G Jayne, R O'Leary, E E Morrison, P J Guillou

**Affiliations:** 1 St James's University Hospital, Academic Unit of Surgery, Clinical Sciences Building, Beckett Street, Leeds LS9 7TF, UK; 2 St James's University Hospital, Cancer Research UK, Clinical Centre Leeds, Beckett Street, Leeds LS9 7TF, UK

**Keywords:** Fas Ligand, Fas, peritoneal, invasion, metastasis

## Abstract

Gastrointestinal carcinomas frequently disseminate within the abdominal cavity to form secondary peritoneal metastases. Invasion of the peritoneal mesothelium is fundamental to this process, yet the underlying invasive mechanisms remain unclear. Preliminary *in vitro* work suggested that tumour cells can induce mesothelial apoptosis, representing a novel mechanism of peritoneal invasion. We examined the role of tumour cell-induced mesothelial apoptosis and explored the role of the death ligand/receptor system, Fas Ligand/Fas, as mediators of the apoptotic process. Cultured human mesothelial cells were used to establish *in vitro* co-culture models with the SW480 colonic cancer cell line. Tumour-induced mesothelial apoptosis was confirmed by phase-contrast microscopy and apoptotic detection assays. Human mesothelial cells and SW480 tumour cells constitutively expressed Fas and Fas Ligand mRNA and protein as determined by RT–PCR and confocal fluorescent microscopy. Stimulation of human mesothelial cells with anti-Fas monoclonal antibody or crosslinked soluble Fas Ligand-induced apoptosis, confirming the functional status of the Fas receptor. Pretreatment of SW480 cells with a blocking recombinant anti-Fas Ligand monoclonal antibody significantly reduced mesothelial apoptosis, indicating that tumour-induced mesothelial apoptosis may, in part, be mediated via a Fas-dependent mechanism. This represents a novel mechanism of mesothelial invasion and offers several new targets for therapeutic intervention.

Despite the peritoneum being a frequent site for the dissemination of primary gastrointestinal cancers, little is known about the mechanisms involved in peritoneal carcinomatosis. Of paramount importance in the peritoneal metastatic cascade is the invasion by tumour cells of the innermost layer of the peritoneum, the mesothelium, a single layer of mesothelial cells. Only when this protective barrier has been breached can tumour cells infiltrate and proliferate within the submesothelial connective tissue matrix.

Several theories have been proposed, which attempt to explain the mechanisms by which tumour cells invade the mesothelium. Early animal studies suggested that invasion of the mesothelium occurred by a process reminiscent of neutrophil extravasation, with adherent tumour cells pushing cytoplasmic projections between mesothelial intercellular junctions (Akedo *et al*, 1986). Although the mechanisms of peritoneal neutrophil extravasation have subsequently been well characterised, and the adhesion molecules involved elucidated (Li *et al*, 1998), little evidence exists to support a similar mechanism in peritoneal metastasis.

Other researchers have suggested that rather than ‘pushing’ their way through the mesothelial monolayer, tumour cells induce mesothelial retraction and dissagregation to gain access to the submesothelial connective tissue. This phenomenon was believed to result from soluble tumour-derived factors as it could be reproduced by either tumour-conditioned media or exogenous inflammatory cytokines (Yonemura *et al*, 1996, 1997).

More recently, it has been suggested that tumour adhesion, rather than the presence of soluble factors, is the prerequisite for mesothelial invasion. It is well documented that tumour cells adhere rapidly to the mesothelium both *in vivo* and *in vitro*, and a role for the cell adhesion molecule CD44 and the integrins *β*1, *α*2, *α*3 and *α*5 in mesothelial invasion has been postulated (Kiyasu *et al*, 1981; Schlaeppi *et al*, 1997; Lessen *et al*, 1999; Strobel and Cannistra, 1999). In these studies, tumour adhesion appeared to precede dissagregation of the mesothelial monolayer, and could be partially blocked by pretreatment with anti-CD44 and anti-*β*1 antibodies.

Previous work in our laboratory using an *in vitro* three-dimensional model of the peritoneum has shown that tumour cells rapidly adhere to human mesothelial cells prior to the initiation of the invasive process (Jayne *et al*, 1999). We have also confirmed that tumour adherence is shortly followed by destruction of the mesothelial monolayer, in a process that involves mesothelial retraction. However, mesothelial retraction is just one component of a more complex cellular response that includes cell shrinkage, nuclear fragmentation, and membrane-blebbing. On the basis of these observations we hypothesised that mesothelial invasion occurred as a result of tumour-induced mesothelial cell apoptosis.

If an apoptotic mechanism is indeed the driving force behind mesothelial invasion, it begs the question as to which mediators of apoptosis are involved. Tumour-induced apoptosis has been implicated in other aspects of cancer biology, in particular as an instrument for evading immunological recognition (Griffith *et al*, 1995; Hahne *et al*, 1996). Central to many of these investigations has been the death ligand/receptor system Fas Ligand (FasL)/Fas, which has been shown to be involved in the apoptotic killing of host immune cells (O’Connell *et al*, 1996). It seems reasonable therefore to explore the possibility that a similar FasL/Fas mechanism may be responsible for executing tumour-induced mesothelial cell death.

The aims of the current study were therefore two-fold. Firstly, to confirm that the changes in mesothelial morphology seen on co-culture with tumour cells are in fact a result of mesothelial cell apoptosis, and secondly, to explore the role of the FasL/Fas system as a potential mediator of mesothelial apoptotic cell death.

## MATERIALS AND METHODS

### Cell culture and characterisation of human peritoneal mesothelial cells

All cell culture equipment and reagents were purchased from GibcoBRL (Life Technologies, Paisley, UK) unless otherwise stated. Human peritoneal mesothelial cells were isolated from omental biopsies obtained from resection specimens of patients undergoing elective abdominal surgery (local ethical committee approved) using a modification of a previously described method (Styliano *et al*, 1990). Briefly, biopsies were washed in sterile 10% Hank's Balanced Salt Solution (HBSS) to remove excess blood contamination, and subjected to enzymatic degradation by incubation in trypsin/HBSS (2.5 mg ml^−1^) at 37°C under continuous agitation for 20 min. Liberated mesothelial cells were pelleted by centrifugation (1000 **g** for 10 min, Mistral 2L, Measuring & Scientific Equipment Ltd, Crawley, UK) and washed in Ham's F-12 culture medium supplemented with 10% Foetal Calf Serum (FCS). Mesothelial cells were resuspended, counted, and plated into 25-cm^3^ tissue culture flasks coated with 0.1% gelatin (Sigma, Gillingham, UK) at 1 × 10^5^ cells ml^−1^. Cells were cultured to confluence (37°C, 95% O_2_/5% CO_2_ atmosphere) in Ham's F12 medium supplemented with 10% FCS, 5 ml penicillin (25 000 IU ml^−1^), 5 ml streptomycin (5000 *μ*g ml^−1^), 50 IU human soluble Insulin (Humulin S, Eli Lilly & Company Ltd, Basingstoke, UK), bovine transferrin (5 *μ*g ml^−1^), and hydrocortisone (0.4 *μ*g ml^−1^, Pharmacia & Upjohn Limited, Milton Keynes, UK). This medium will subsequently be referred to as Ham's F12 complete medium. Media was changed every 3 days. Mesothelial cultures were characterised by indirect immunofluorescent cytochemistry for cytoskeletal proteins cytokeratin 8, 18, and vimentin, with contaminating endothelial and tumour cells excluded by an absent expression of von Willibrand Factor and BerEp4 antigens respectively. All experiments were performed on cultures of passage 3 or less to avoid spurious results due to *in vitro* phenotypic degeneration.

The human primary colonic cell line, SW480, was purchased from the European Collection of Cell Cultures (ECCAC, Porton Down, UK) and maintained in RPMI 1640 medium with L-glutamine, supplemented with 10% FCS, 5 ml penicillin (25 000 IU ml^−1^) and 5 ml streptomycin (5000 *μ*g ml^−1^).

### Characterisation of tumour-induced mesothelial apoptosis

Mesothelial cells were seeded onto 1% gelatin coated glass coverslips in six-well culture trays at 1 × 10^5^ cells ml^−1^ and grown to confluence in Ham's F-12 complete media at 37°C in an atmosphere of 95% O_2_/5% CO_2_. SW480 tumour cells were passaged, their viability tested by trypan-blue dye exclusion, and counted in an Improved Neubauer chamber. In total, 1 × 10^4^ tumour cells/ml in RPMI-1640 supplemented media were seeded onto the mesothelial monolayers and incubated at 37°C in an atmosphere of 95% O_2_/5% CO_2_. Changes in mesothelial morphology in the co-cultures were recorded by serial time course photomicroscopy.

Similar co-culture models were set up for dual-antigen immunocytochemistry. Mesothelial apoptosis was detected in the co-culture models by DNA fragmentation assay (Klenow-FragEL™, Calbiochem, Nottingham, UK), according to the manufacturer's instructions. SW480 tumour cells were identified by immunocytochemical labelling with mouse anti-human BerEp4 antibody (1 : 200 in PBS+10% rabbit serum; Dako, Cambridgeshire, UK), followed by a biotinylated rabbit anti-mouse secondary antibody (1 : 500 in PBS+10% rabbit serum, Dako), and visualised using the alkaline phosphatase-streptavidin/Fast Red™ system (Sigma, Poole, UK). All slides were counterstained with Harris's haematoxylin, and mounted in Aquamount™ (BDH, Poole, UK). Images were visualised with a Leitz™ E1000 light microscope and recorded with Lucia™ image-analysis software.

### Co-culture model and quantification of mesothelial apoptosis

Mesothelial cells were grown to confluence on 1% gelatin coated glass coverslips in Hams’ F12 complete medium, and seeded with SW480 at an effector : target ratio of 1 : 1. Cellular interactions were terminated after 8 h incubation by fixation with 3.5% paraformaldehyde in buffered saline at 4°C for 15 min, and mesothelial apoptosis determined by DNA fragmentation assay using the *In situ* Cell Death Detection Kit, Alkaline Phosphate (AP) (Boehringer Mannheim Gmbh, Mannhein, Germany) as per manufacturers instructions. Briefly, fixed apoptotic cells were permeabilised with 0.1% Triton X-100 in 0.1% sodium citrate for 2 min at 4°C, and subjected to TdT-mediated dUTP nick end fluorescent labelling (TUNEL). Incorporated fluorescein was detected using an antifluorescein Fab antibody conjugated with alkaline phosphatase, and visualised by incubation with Fast Red™ for 10 min at room temperature. The mesothelial apoptotic index (AI) was calculated by counting the number of apoptotic cells in 10 random high power fields (× 200 magnification) using image-analysis software as previously described (Lipponen *et al*, 1994). Apoptotic mesothelial cells were readily differentiated from any contaminating apoptotic tumour cells by their position within the focal plane of the microscope.

Positive controls included fixed permeabilised cells incubated with DNAse 1 (Sigma) at 1 mg ml^−1^ in 50 mM Tris-HCl, pH 7.5, 1 mg ml^−1^ BSA for 10 min at room temperature. Omission of the terminal transferase enzyme acted as a negative control. Mesothelial monolayers and tumour cultures on their own were used as sample controls. All experiments were repeated in triplicate.

To determine the potential involvement of soluble tumour derived apoptotic factors, mesothelial monolayers were incubated with different concentrations of preconditioned medium from SW480 cultures.

### Fas/FasL mRNA expression in SW480 and human mesothelial cells

Total RNA was isolated from individual samples of SW480 and human mesothelial cells using TRIzol® Reagent (Life Technologies) as per manufacturer's instructions, and 10 *μ*l of each reverse transcribed using 120 U of MMLV-reverse transcriptase (Promega, Southampton, UK), 0.5 *μ*g of oligo d(t)_18_ primers, 1 mmol of each dNTP's (Amersham Biosciences U.K. Limited, Chalfont St Giles, UK), 20 U of RNAsin (Promega), 5 × reverse transcriptase reaction buffer (250 mmol Tris-HCl, (pH 8.3), 375 mmol KCl, 15 mmol MgCl_2_ and 50 mmol DTT, Promega) to a total volume of 20 *μ*l. Reverse transcription was performed using a thermal program of 42°C for 60 min and 95°C for 10 min. PCR was performed by the addition of 1 *μ*l of cDNA to a 20 *μ*l reaction mixture containing 10 × Taq reaction buffer (500 mM KCl, 100 mM Tris-HCl (pH 9.0) and 1% Triton® X-100, Promega), 20 pmol of each primer, 1 U of *Taq* Polymerase (Promega) and 0.2 mM dNTPs. The mixture was overlaid with 50 *μ*l of mineral oil to prevent evaporation. PCR was initiated in a thermal cycler ((Biometra TRIO-Thermoblock, Gõttingem, Germany) and programmed for 35 cycles of 1 min at 95°C, 1 min at 55°C, 1 min at 72°C, and a final elongation step of 5 min at 72°C. Gene-specific primers were designed to span at least one intron and included: human FasL, forward primer 5′-dCAGCTCTTCCACCTACAGAAGG-3′ (bp 464–485) and reverse primer 5′-dAAGATTGAACACTGCCCCCAGG-3′ (bp 889–910), resulting in a product of 447 bp length; human Fas, forward primer 5′-dCAAAGCCCATTTTTCTTCCA-3′ (bp 499–518) and reverse primer 5′-dGACAAAGCCACCCCAAGTTA-3′ (bp 755–774), resulting in a product of 276 bp length; and human GAPDH as the house-keeper gene, forward primer 5′-dGAGTCAACGGATTTGGTCGT-3′ (bp 53–72) and reverse primer 5′-dGGTGCCATGGAATTTGCCAT-3′ (bp 191–210), resulting in a product of 158 bp length. PCR products were resolved by electrophoresis through 2% agarose gels and visualised by ethidium bromide under UV fluorescence.

Human T-cells were isolated from healthy volunteers by standard Ficoll density gradient centrifugation (Lymphoprep™, Nycomed Pharma AS, Drammensveien, Norway), stimulated with 1 *μ*g ml^−1^ of phorbol 12-myrisate 13-actetate (Sigma) for 48 h, and used as control samples for Fas and FasL expression in the PCR reactions.

### Determination of functional activity of FasL/Fas

To investigate the functional status of the Fas receptors, mesothelial monolayers were incubated with either soluble FasL recombinant protein (2.5 ng ml^−1^) and enhancer (1 *μ*g ml^−1^) (Alexis Corporation, Nottingham, UK) or agonistic anti-Fas monoclonal antibody 2R2 (1 *μ*g ml^−1^) (Alexis Corporation) for 0, 2, 4, 6, 8, and 12 h. Mesothelial apoptosis was quantified as above using the *In Situ* Cell Death Detection Kit (Boehringer Mannheim).

To investigate the functional role of FasL/Fas interaction, SW480 FasL expression was blocked by preincubating SW480 cells with a FasL inhibiting recombinant protein, which consisted of the extracellular domain of human Fas fused to the Fc portion of Human IgG1 (rhFas:Fc) (Alexis Corporation) at an optimum concentration of 5 *μ*g ml^−1^ and enhancer protein 1 *μ*g ml^−1^ for 60 min at 37°C. The effect of tumour FasL blockade on mesothelial apoptosis was quantified using the co-culture model as described above.

### Localisation of FasL/Fas expression by immunofluorescent confocal microscopy

Investigation of SW480 and mesothelial FasL/Fas interaction was performed on fixed co-cultures using a dual immunofluorescent antigen detection technique. Samples were incubated with blocking solution (PBS supplemented with 10% goat and donkey serum) for 60 min, rinsed with PBS, and incubated with a primary rat monoclonal anti-human FasL specific IgG antibody (Mike-1, Alexis Corporation) at 1 *μ*g ml^−1^ and a primary mouse monoclonal anti-human Fas specific IgG antibody (APO-1, Dako) at 0.15 *μ*g ml^−1^. Visualisation was performed using a TRITC conjugated goat anti-rat IgG secondary antibody (Chemicon International, Harrow, UK) at 0.5 *μ*g ml^−1^ and a FITC-conjugated donkey anti-mouse IgG secondary antibody (Chemicon International) at 0.5 *μ*g ml^−1^ for FasL and Fas respectively. All samples were mounted in Vectashield (Vecta Laboratories, CA, USA) and viewed with a laser scanning confocal microscope (Lieca TCS-SP). Three-dimensional image rotations were derived from confocal image stocks using the confocal system software. Control samples were exposed to blocking solution and secondary antibody alone.

### Statistical analysis

Differences in apoptotic indices between the co-culture groups were tested using the Mann–Whitney *U*-test, with a significance level of *P*<0.05.

## RESULTS

### Tumour cells-induced mesothelial apoptosis

**Figure 1:**
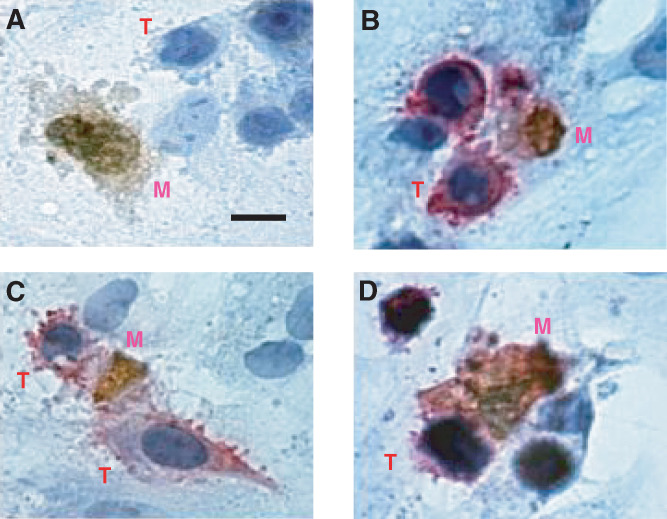
Mesothelial-SW480 co-cultures. The tumour cells, T, induce apoptosis in adjacent mesothelial cells, M. Mesothelial DNA fragmentation is apparent as brown nuclear staining. Cell shrinkage and characteristic membrane blebbing are apparent in (**A**) (Tumour cells unstained in this experiment). (**B–D**), Dual staining of mesothelial-SW480 co-cultures. Tumour cells (T) are identified by their position in the focal plane and by their positive membrane staining (red) for BerEp4 epithelial antigen. Adjacent mesothelial cells (M) show apoptotic changes with nuclear fragmentation (brown). Bar, 2.5 *μ*m.

Phase-contrast microscopy of SW480-mesothelial co-cultures showed that mesothelial cells adjacent to adherent tumour cells underwent a change in cellular morphology with cytoplasmic shrinkage, membrane blebbing, and nuclear fragmentation, characteristic of apoptotic cell death (Figure 1

). Time course experiments using the co-cultures and TUNEL assay revealed that mesothelial apoptosis occurred within hours of tumour seeding, increased to a maximum by 8 h, and returned to basal levels by 24 h.

### FasL/Fas mRNA expression in SW480 and human mesothelial cells

RT–PCR analysis showed that human mesothelial cells constitutively expressed Fas mRNA but not FasL mRNA. SW480 cells expressed both FasL and Fas mRNA.

### Mesothelial cells express functional Fas receptor

**Figure 2:**
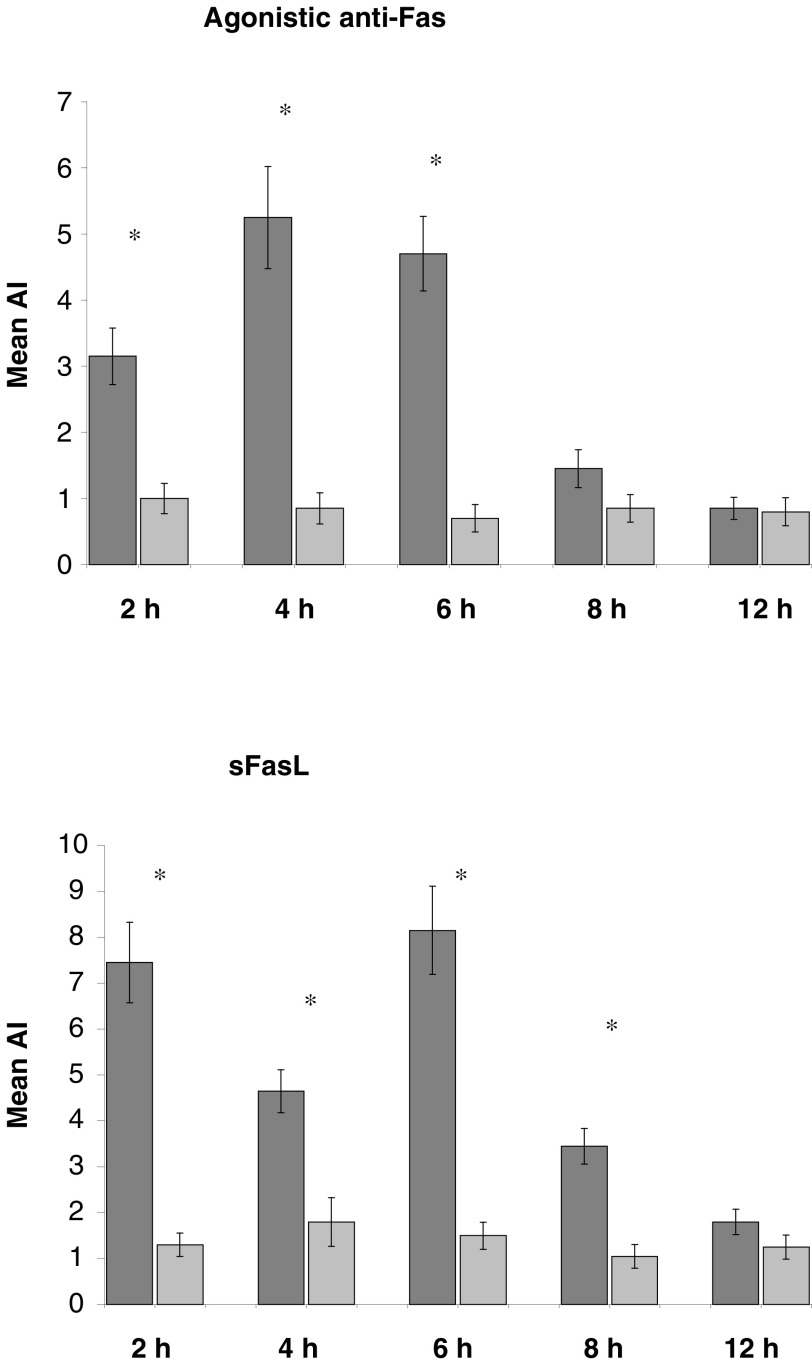
Assessment of the functionality of human mesothelial Fas. Mesothelial monolayers were incubated with an agonistic anti-Fas mAb or stimulating crosslinked sFasL (dark columns). Controls were untreated mesothelial monolayers (light columns). Mesothelial apoptosis was detected using a TUNEL assay. Results are expressed as mean AI of triplicate experiments±s.d. ^*^P<0.05, Mann–Whitney *U*-test.

Pretreatment with either agonistic anti-Fas antibody or stimulating crosslinked human FasL resulted in mesothelial cell death, which was maximal at 4 and 6 h respectively, as compared to untreated controls (Figure 2

).

### SW480 Tumour cells induce Fas-mediated apoptosis of mesothelial cells

**Figure 3:**
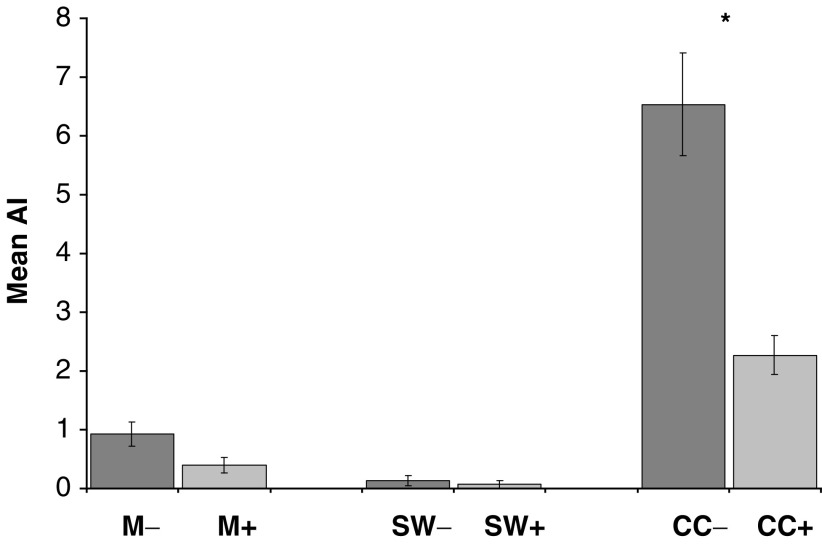
Quantitative co-culture apoptosis assays. SW480 cells were pretreated with a FasL inhibiting recombinant protein rhFas:Fc prior to addition to mesothelial monolayers as co-cultures (CC). Monolayers (M) and SW480 tumour cells (SW) were cultured alone as controls. ± denotes pretreatment of cultures with recombinant protein. All co-culture experiments were terminated at 8 h. Mesothelial cell death is expressed as mean AI of triplicate experiments±s.d. ^*^P<0.05, Mann–Whitney *U*-test.

Co-culture of SW480 cells with mesothelial monolayers increased the mean mesothelial AI by a factor of 700% compared to controls (Figure 3

, CC− *vs* M−, *P*<0.001). Control cultures of mesothelial monolayers alone showed a low basal rate of apoptosis. Negligible tumour cell apoptosis was detected either in the co-cultures or in SW480 cells cultured alone. Incubation of mesothelial monolayers with SW480 preconditioned media of various concentrations and for the same time points failed to significantly induce mesothelial apoptosis.

Pretreatment of SW480 tumour cells with monoclonal anti-human recombinant FasL reduced mesothelial apoptosis in co-culture models by 290% compared to non-pretreated controls (Figure 3, CC− *vs* CC+, *P*<0.001). However, the mesothelial AI in pretreated samples was still significantly greater than the basal levels recorded in pure mesothelial cultures (Figure 3, CC+ *vs* M−, *P*<0.001). Treatment with anti-human FasL had no effect on mesothelial monolayers or tumour cells cultured separately.

### Localisation of Fas/FasL expression by immunofluorescent confocal microscopy

**Figure 4:**
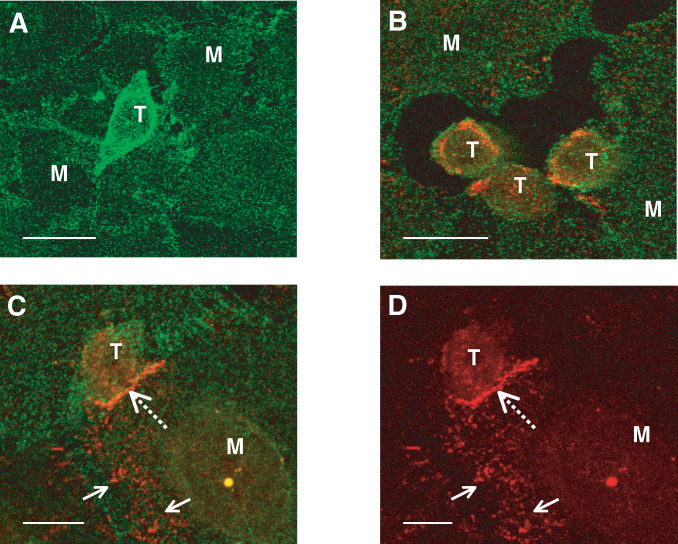
FasL/Fas interaction in tumour-mediated mesothelial apoptosis. Dual confocal fluorescent microscopy of co-cultures with anti-FasL and anti-Fas antibodies. FasL and Fas were visualised with a TRITC and FITC secondary antibody respectively. Mesothelial cells demonstrate homogenous cell surface expression of Fas (**A**) and undergo cytoplasmic retraction following tumour cell interaction (**B**). Tumour cell FasL is polarised at areas of mesothelial contact (**C**, broken arrow). FasL is not constitutively expressed by mesothelial cells but is induced by the presence of adherent tumour cells (**C**, arrows). FasL channel only (**D**). M, mesothelial cell. T, SW480 tumour cell. (**A**) Bar 20 *μ*m; (**B**) bar 20 *μ*m; (**C**) bar 5 *μ*m; and (**D**) bar 10 *μ*m.

Immunofluorescent staining revealed a homogeneous distribution of cell membrane Fas expression in mesothelial cells (Figure 4A

), but no constitutive FasL expression. Mesothelial cell retraction was seen in the vicinity of invading tumour cells, which expressed both Fas and FasL protein (Figure 4B). In areas of tumour–mesothelial adhesion, tumour FasL became polarised to areas of cell-to-cell contact (Figure 4C and D), while mesothelial cell Fas expression remained homogenous. Initially, the polarisation of tumour–FasL was thought to be indicative of receptor–ligand interaction. However, confocal studies using three-dimensional reconstruction and detailed examination of individual confocal sections were unable to confirm FasL/Fas co-localisation. Interestingly, mesothelial expression of FasL, although not constitutively expressed, was markedly induced in cells adherent with tumour cells (Figure 4C and D). Incubation of pure mesothelial cultures with Fas-receptor activators (agonistic anti-Fas mAb or crosslinked sFasL) failed to upregulate mesothelial FasL mRNA expression on semiquantitative PCR analysis (results not shown), indicating that increased transcriptional activity was unlikely to account for the mesothelial expression of FasL membrane protein seen in the co-culture models.

## DISCUSSION

We have demonstrated for the first time, using an *in vitro* model, that a colorectal cancer cell line is capable of inducing human mesothelial cell apoptosis. Although previous authors have reported changes in mesothelial morphology, namely cytoplasmic retraction, following intraperitoneal injection of tumour cells or cytokines, the significance of these observations appears to have gone unnoticed (Akedo *et al*, 1986). Using phase-contrast microscopy and apoptosis detection assays we have shown that the observed cellular retraction is but one component of a more complex process that includes membrane-blebbing and nuclear fragmentation, which are the hallmarks of apoptotic cell death. We therefore propose that tumour-induced mesothelial apoptosis is an important mechanism by which disseminated tumour cells breach the mesothelium to gain access to the peritoneal connective tissue.

Previous authors have suggested that soluble tumour-derived factors may be responsible for inducing mesothelial retraction. However, these studies were performed in animal models using high inoculations of tumour cells or unphysiological concentrations of inflammatory cytokines. Contrary to these findings, in human mesothelial cells we have demonstrated that tumour–mesothelial adhesion is required for the induction of apoptosis, which cannot be reproduced by the substitution of tumour-conditioned media. This is supported by other *in vitro* work that has implicated cell adhesion molecules, such as CD44 and the *β*1 integrins, in tumour-mesothelial invasion, (Schlaeppi *et al*, 1997; Lessen *et al*, 1999; Strobel and Cannistra, 1999). The requirement for cell–cell contact to initiate the invasive process supports the role of cell membrane bound ligands, such as FasL, as the mediators of mesothelial apoptosis. FasL, although primarily expressed in cells of lymphoid lineage (Suda *et al*, 1993) and in immune privileged sites (Bellgrau *et al*, 1995), has also been shown to be expressed by a number of carcinomas, including colorectal (Shiraki *et al*, 1997), hepatocellular (Strand *et al*, 1996) and lung (Niehans *et al*, 1997). Furthermore, FasL is more frequently expressed in colorectal liver metastases than in matched primary carcinomas (Mann *et al*, 1998). The expression of FasL would therefore appear to confer a selective advantage to metastasising tumour cells.

Our RT–PCR and fluorescent immunocytochemistry data demonstrates that SW480 carcinoma cells express FasL. This has been previously documented in the SW480 cell line in addition to other carcinoma cell lines (Shiraki *et al*, 1997; Mann *et al*, 1998; O’Connell *et al*, 1998). Importantly, we have also shown that human mesothelial cells constitutively express cell-surface Fas, a previously undocumented finding, and that the mesothelial Fas receptor is functionally active. Blocking of SW480 FasL activity with a recombinant anti-FasL significantly reduced mesothelial apoptosis in our co-culture model and supports the role of the Fas death ligand/receptor system in mesothelial invasion. This mechanism is analogous to that described by O’Connell *et al* (1996) who showed that the colonic cell line, SW620, induces T-cell apoptosis in a ‘counterattack’ mechanism of immune evasion via engagement of tumour FasL and T-cell Fas. Similarly, immune cell FasL has been implicated in the apoptotic cell death of hepatocytes in viral hepatitis (Mita *et al*, 1994) and in the *in vitro* death of hepatocytes by FasL expressing colon cancer cell lines (Yoong *et al*, 1999).

Further indirect evidence of FasL/Fas involvement in mesothelial apoptosis is provided by our confocal microscopy studies. Tumour FasL expression was localised to areas of tumour–mesothelial adhesion suggesting its involvement in cell–cell signalling. Unfortunately, we were unable to demonstrate tumour FasL–mesothelial Fas co-localisation, which would have provided strong proof of a FasL/Fas interaction at this site. Two possible explanations exist for this. Firstly FasL/Fas interactions might occur as rapid events, in low densities, making it difficult to demonstrate at an isolated time point. Alternatively, individual tumour cells from a heterogeneous cell line may utilise other death ligands such as TRAIL. Certainly, FasL/Fas signalling is unlikely to be the sole mechanism initiating mesothelial apoptosis, as pretreatment of SW480 cells with recombinant anti-Fas only partially reduced mesothelial apoptosis.

Restifo *et al* (2000) have disputed the FasL/Fas tumour ‘counter-attack’ mechanism as being over-simplistic, while others (Zaks *et al*, 1999) have demonstrated that in tumour–immune cell interactions, lymphocytes initially recognise tumour cells via MHC/T-cell receptor complexes and subsequently upregulate their FasL expression. This in turn induces Fas-mediated apoptosis in adjacent lymphocytes in a paracrine manner. We have seen a similar upregulation of mesothelial FasL expression in those cells in contact with adherent tumour cells by confocal microscopy. This increased FasL protein expression could not be reproduced at the mRNA level by stimulation with exogenous Fas receptor agonists. It is therefore possible that mesothelial surface expression of FasL protein resulted from either its rapid release from intracellular stores (Martinez-Lorenzo *et al*, 1999) or by transcriptional upregulation induced by non-Fas mediated signalling, for example by TRAIL or TNF-*α* (Herr *et al*, 2000).

In summary, we believe tumour-induced mesothelial apoptosis is an important mechanism in the process of peritoneal invasion. By removing the defensive mesothelial barrier and allowing exposure of adherent tumour cells to the underlying submesothelial connective tissue, this mechanism represents an important first step in the peritoneal metastatic cascade. The existence of functional mesothelial Fas receptors, and the ability to inhibit tumour-induced apoptosis by the use of blocking anti-FasL proteins, suggests a role for these death ligands and receptors as the mediators of mesothelial apoptosis. The intricacies of mesothelial Fas signalling and, in particular, the significance of upregulation of mesothelial FasL in the vicinity of adherent tumour cells, remains an area of ongoing research. Further *in vivo* investigations using animal models would be of value to corroborate our findings for the role of FasL/Fas and to test its efficacy as a possible therapeutic target. Of importance is the discovery of a novel mechanism underlying peritoneal metastasis, and with it the potential to identify and develop novel and more specific therapeutic strategies to combat peritoneal carcinomatosis of gastrointestinal origin.
